# Early hematopoietic stem cell transplantation improve treatment outcome of aggressive ATL.

**DOI:** 10.1186/1742-4690-12-S1-P28

**Published:** 2015-08-28

**Authors:** Kaoru Uchimaru, Nobuhiro Ohno, Seiichiro Kobayashi, Ryuji Tanosaki, Shigeo Fuji, Takahiro Fukuda, Arinobu Tojo

**Affiliations:** 1 Department of Hematology/Oncology, Research Hospital, Institute of Medical Science, the University of Tokyo, Tokyo, Japan; 2 Department of Molecular Therapy, Institute of Medical Science, the University of Tokyo, Tokyo, Japan; 3 Department of Blood Transfusion and Cellular Therapy, National Cancer Center Hospital, Tokyo, Japan; 4 Hematopoietic Stem Cell Transplantation Division, National Cancer Center Hospital, Tokyo, Japan

## 

Aggressive ATL is one of the most intractable peripheral T cell malignancies and outcome of chemotherapy is still unsatisfactory. Hematopoietic stem cell transplantation (HSCT) is a promising strategy to improve treatment results of aggressive ATL but the proportion of the ATL patients who received HSCT in the potential candidates is low. In this study we treated 45 consecutive aggressive ATL patients with intensive combination chemotherapies (mainly mLSG15) followed by HSCT if indicated. We started to search HSC donor as soon as possible after starting chemotherapy in order to advance HSCT. In a total of 45 patients, 39 were acute type, 4 were lymphoma type, and 2 were unfavorable chronic type. The median age of the patients was 59 years (range 28-68). Thirty-one patients finally received allo-HSCT (related BMT/PBSCT 5, unrelated BMT 26). The proportion of the patients who received HSCT was 68.9%. Disease status at transplantation was CR in 2, PR in 17, SD in 7 and PD in 4 patients. Median time to HSCT from beginning of chemotherapy was 166 days. Three-year overall survival (OS) of the all patients receiving HSCT and those who receive HSCT in CR/PR were 60.0% and 76.5% respectively. Three-year OS of total 45 patients was 41.5%. Among 13 patients who could not receive HSCT, 6 relapsed after initial response to chemotherapy (median time to relapse from the beginning of chemotherapy was130 days) and 2 were in primary refractory state, which were the reason they could not receive HSCT. Those 6 relapsed patients might receive HSCT if they could find suitable HSC donor earlier. Our study suggest that early surveillance of HSCT donor along with remission induction chemotherapy raise up the proportion of patients receiving HSCT and improve treatment results in aggressive ATL.

